# Chicken Astro virus (CAstV): Isolation and characterization of new strains in broiler flocks with poor performance

**DOI:** 10.1007/s11259-023-10109-x

**Published:** 2023-03-28

**Authors:** Ahmed A. Sallam, Asmaa K. Al-Mokaddem, Mohamed M. Hamoud, Mahmoud Samir, Rabab Amin Khalifa, Sherein S. Abdelgayed

**Affiliations:** 1 Broiler sector, CPC, Americana Group, Cairo, Egypt; 2 Department of Pathology, Faculty of Veterinary Medicine, Cairo University, Giza, 12211 Egypt; 3 Department of Poultry and Rabbit Disease, Faculty of Veterinary Medicine, Cairo University, Giza, Egypt; 4 Reference Laboratory for Veterinary Quality Control On Poultry Production- Animal Health Research Institute (AHRI), Agriculture Research Centre (ARC), Dokki, Giza, Egypt; 5 Cairo Poultry Company (CPC) Laboratory Division, Cairo, Egypt

**Keywords:** Chicken Astrovirus, New isolates, Histopathology, Bird performance

## Abstract

Chicken astroviruses (CAstV) were associated with retarded growth, enteritis, kidney diseases, and white chick syndrome. In the current study, we aimed to evaluate the effect of CAstV infection on growth, performance, and gross and histopathological picture of commercial chicken flocks suffering increased culling rate and decreased performance. Samples were collected for virus isolation, identification, and sequencing on day one, 15 days, and 30 days of age. Body weight, feed conversion rate, and mortality rates were determined. A gross examination was performed, and tissue samples from the liver, intestine, kidneys, heart, and lungs were kept in formalin for histopathological evaluation. Embryos inoculated with CAstV revealed dwarfism, and edema. The cytopathic effect on CAstV inoculated cells included aggregation,, and sloughing. The isolated Egyptian isolates shared the highest nucleotide homology (93%) with the Korean isolate Kr/ADL102655-1/2010 and showed the most distant relation to the Indian isolate Indovax/APF/1319 with 82–83% homology. Body weight exhibited significant reduction with a decrease in feed conversion rate in CAstV infected flocks. Gross examination of CAstV-infected chickens revealed white feathered chicks on day one, and poor body condition in older chickens as well as swollen kidneys. Histopathological examination of CAstV-infected birds showed mild proventriculitis, shortening of intestinal villi, enteritis, focal hepatocellular necrosis, pericarditis, myocarditis, and proliferative response in lung tissue. Kidneys showed interstitial nephritis, urate deposition, and glomerular hypercellularity. CAstV is a chicken pathogen that could be related to decreased performance, and screening of flocks for CAstV might be an essential step for breeders.

**Supplementary Information:**

The online version contains supplementary material available at 10.1007/s11259-023-10109-x.

## Introduction

In many developing countries, poultry farming is an important food production sector. Poultry production profitability is highly dependent on the health status of the flock. Despite applied biosecurity and intense vaccination programs, an unexpected "failure" may occur. The cause of some problems has remained unknown, but more studies were performed to determine their etiological factors, for example, enteritis-causing factors, including Astroviruses lately (Sajewicz-krukowska et al. 2016).

Astroviruses are small, round, non-enveloped, positive sense-RNA viruses measuring 28–30 nm in diameter. The name comes from their morphology by electron microscopy that resembled the star = 'astron' (Greek for star). Astroviruses cause problems such as retarded growth and gastroenteritis. (Wit et al. 2011).

To date, two different astroviruses have been described in chickens. Avian nephritis virus (ANV) was identified as the first astrovirus of chickens in 2000 (Imada et al. 2000). The other astrovirus, named CAstV, was observed in broiler chickens suffering runting problems in the Netherlands (Baxendale and Mebatsion 2004; Smyth et al. 2012).

Several avian species could be affected by astroviruses specific to a host species for which they are often named (for example, Chicken astrovirus (CAstV). However, cross-infection between host species is known to occur. Avian astroviruses affect young birds causing growth retardation, and vertically transmitted astroviruses can cause 'white Chick Syndrome'. Older birds can be infected but are generally less susceptible to viruses (Smyth 2020).

Astroviruses have been associated with enteritis, hepatitis, nephritis, gout, and runting-stunting syndrome (RSS) in chickens, turkeys, and several duck species (Long et al. 2018).

The current study aimed to compare between a negative CAstV chicken flock and a positive CAstV chicken flock in terms of performance, growth parameters, gross and histopathological changes.

## Material and methods

### Sampling

Thirty cloacal swabs samples were collected in the hatchery on day one to identify CAstV infected and negative flocks by real time-PCR. After gross examination, tissue samples (Proventriculus, intestine, liver, kidney, heart, and lungs) were collected from both chicken flocks (Normal and CAstV positive flocks) on the 1^st^, 15^th^, and 30^th^ days and kept in 10% neutral buffered formalin. CAstV affected birds usually exhibited decreased body weight, growth retardation and general weakness. Other enteric viruses and bacterial infections were excluded by routine screening of the flocks.

### Virus isolation and identification

#### Inoculation in ECE

Five SPF ECE (Specific Pathogen Free Embryonated Chicken Eggs) were inoculated through the intra/yolk route at 5–7 days old with 0.2 ml of the virus sample (Swayne et al. 1998). Additional five eggs were mock inoculated with 0.2 ml PBS. Eggs were candled daily, and mortalities on the first day were discarded and considered non-specific death. On the fifth-day post-inoculation, all remaining eggs were chilled at 4° C overnight. Necropsies were performed for the embryo, and the yolk was harvested and aliquoted for the next passages.

#### Preparation of chicken embryo liver (CEL) cells

CEL cells were prepared from 14- 16 days old specific pathogen-free (SPF) chicken embryos according to Soumyalekshmi et al. (2014) with minor modifications. In brief, the livers were removed and minced into small pieces, then washed with phosphate-buffered saline (PBS) with constant stirring three times (five minutes per each wash). This was followed by three successive trypsinization cycles (five minutes each). The supernatant was collected following each cycle on a complete growth medium and then eventually centrifuged at 1500 rpm for 10 min at 4° C. Cells were seeded at an initial cell count of 1 × 10^6^ for a T25 flask and cultured with DMEM supplemented by 10% fetal bovine serum and 1% penicillin–streptomycin antibiotic. The flasks were incubated at 37.5° C with 5% CO_2_.

#### Isolation of chicken astrovirus (CAstV)

Samples were filtered using a 0.2 µm filter, and 0.5 ml of each sample was inoculated into T25 flasks at 90% confluency. The flasks were incubated at 37.5° C for one hour as adsorption time. This was followed by adding a 10 mL maintenance medium (DMEM supplemented by 1% FBS). The negative control flask also had a medium change, and all flasks were incubated at 37.5° C. Cytopathic effect (CPE) was observed daily.

#### Confirmation of viral isolation

The viral isolation was confirmed by detecting viral nucleic acid using the molecular techniques of PCR and RT–PCR using Kylt® Chicken Astrovirus RT-qPCR according to the manufacturer's recommendations. The Kylt kit can detect both group A and B Chicken Astrovirus. The viral nucleic acids in collected samples were extracted using a QIAamp mini elute spin kit (Qiagen, GmnH, Germany) following the manufacturer's instructions. The RT-PCR for CAstV was performed as previously described (Smyth et al. 2009). The conventional PCR was applied to the extracted RNA using specific primers for the RDRP gene of chicken astrovirus (Table 1). The cDNA was prepared by a commercial kit (RevertAid First Strand cDNA Synthesis Kit, Thermo Scientific) following the manufacturer's instructions.

**Table 1:** Primers located in the hexon gene for gene sequencing

Primer	Sequence	Tm	Reference
Fast-deg-2	5'- GCA TGG CTC CAC CGT AAG C -3'	63.5° C	Smyth et al. 2009
Rast-2	5'- ACA CTC CCA GCA ACA TTT G -3'	58.7° C	This study

#### Gene sequencing

The amplified PCR products with the appropriate size were subsequently purified using a QIAquick Gel Extraction Kit (QIAGEN, Hilden, Germany). The purified PCR products were directed for sequencing reactions using a Big Dye Terminator v3.1 Cycle Sequencing Kit (Applied Biosystems, Foster City, CA); according to the manufacturer, the reaction product was purified by exclusion chromatography in DyeEX 2.0 Spin Kit. The recovered materials were sequenced using a 3500 XL DNA Analyzer (Applied Biosystems).

The four partial sequences of this study were identified as CAstV/ORF1b (corresponds to RDRP gene) by a basic local alignment search tool (BLAST) search (https://blast.ncbi.nlm.nih.gov/Blast.cgi) and compared with the published CAstV deposited in the GenBank database. The nucleotide sequence of each of the four Egyptian isolates was deposited to GenBank under the accession numbers shown in Table 2. Next, the sequences were aligned and analyzed with other related sequences in the GenBank database. The nucleotide and deduced amino acid sequences of ORF1b obtained in this study were aligned using the Clustal W methods in Bioedit software (Hall 1999). The similarity percentages were calculated using a sequence identity matrix for nucleotides and amino acids. The phylogenetic tree of the four Egyptian isolates was constructed using the software package MEGA X (Kumar et al. 2018).

**Table 2:**
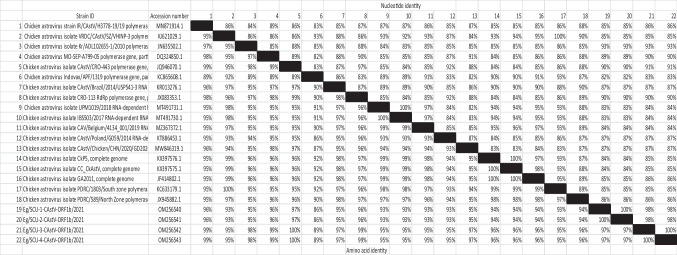
Nucleotide and amino acid similarity between the Egyptian isolates and global strains of CAstV

### Clinical signs, growth, performance, and mortality rate

Clinical signs were observed in normal and CAstV-infected flocks (real time-PCR positive for CAstV).The means of body weight, feed conversion rate (FCR), and mortality rate were recorded in the studied houses including five normal and five CAstV-positive flocks (each contained 1000 birds of known source) to be compared with the species standard indices.

### Gross and histopathological examination

On the 1^st^, 15^th^, and 30^th^ days, thirty birds were collected for postmortem examination, and tissue samples from proventriculus, intestines, liver, kidneys, heart, and lungs were kept in 10% neutral buffered formalin, routinely processed and stained with hematoxylin and eosin (H&E) for light microscopy (Bancroft and Gamble 2008).

### Statistical analysis

Data are shown as the mean ± standard error (SEM). The significance was considered at *P*<0.05 using one way ANOVA test using GraphPad Prism 8 software.

## Results

### Virus isolation

**Fig. 1:**
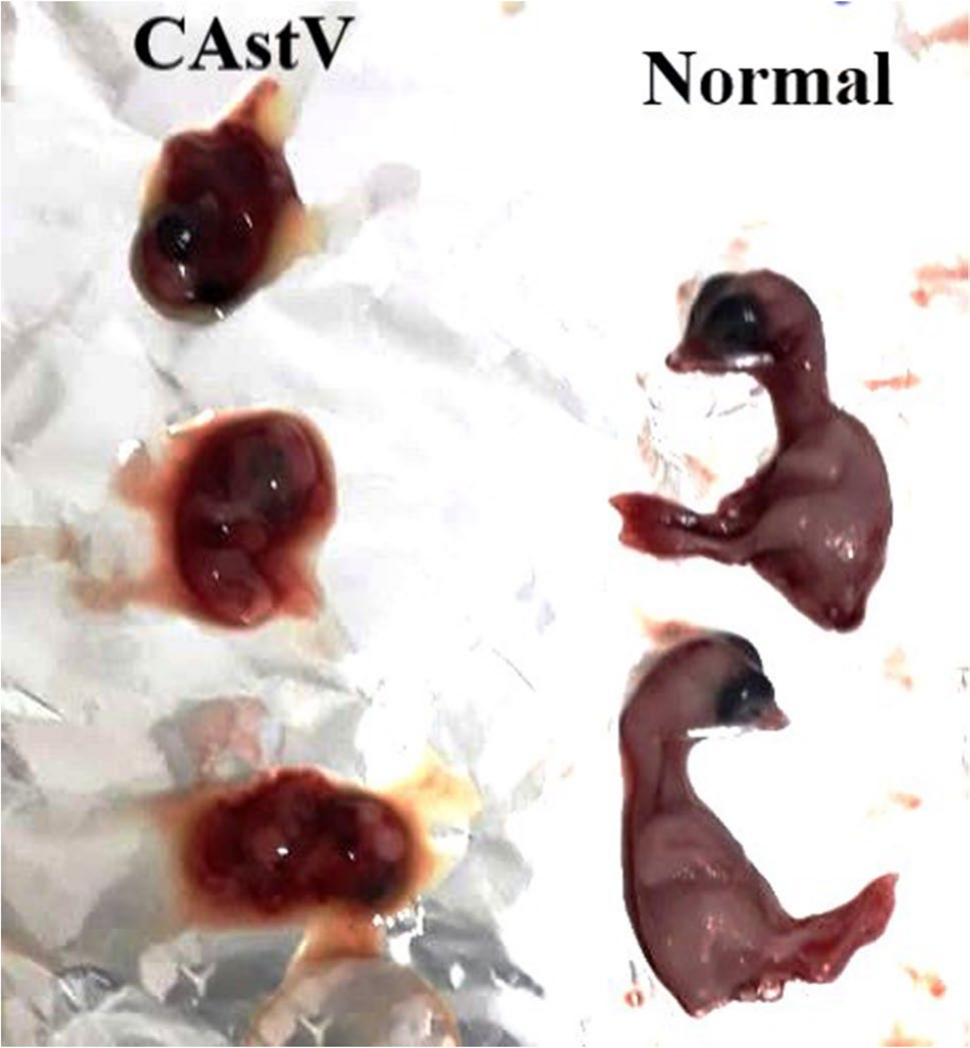
Chicken embryos showing normal healthy embryo and dwarfism, hemorrhage, and edema in CAstV infected embryo

The embryos showed lesions, including severe hemorrhages, edema, and dwarfism. Meanwhile, negative control embryos showed healthy embryos with no lesions (Fig. 1).

**Fig. 2:**
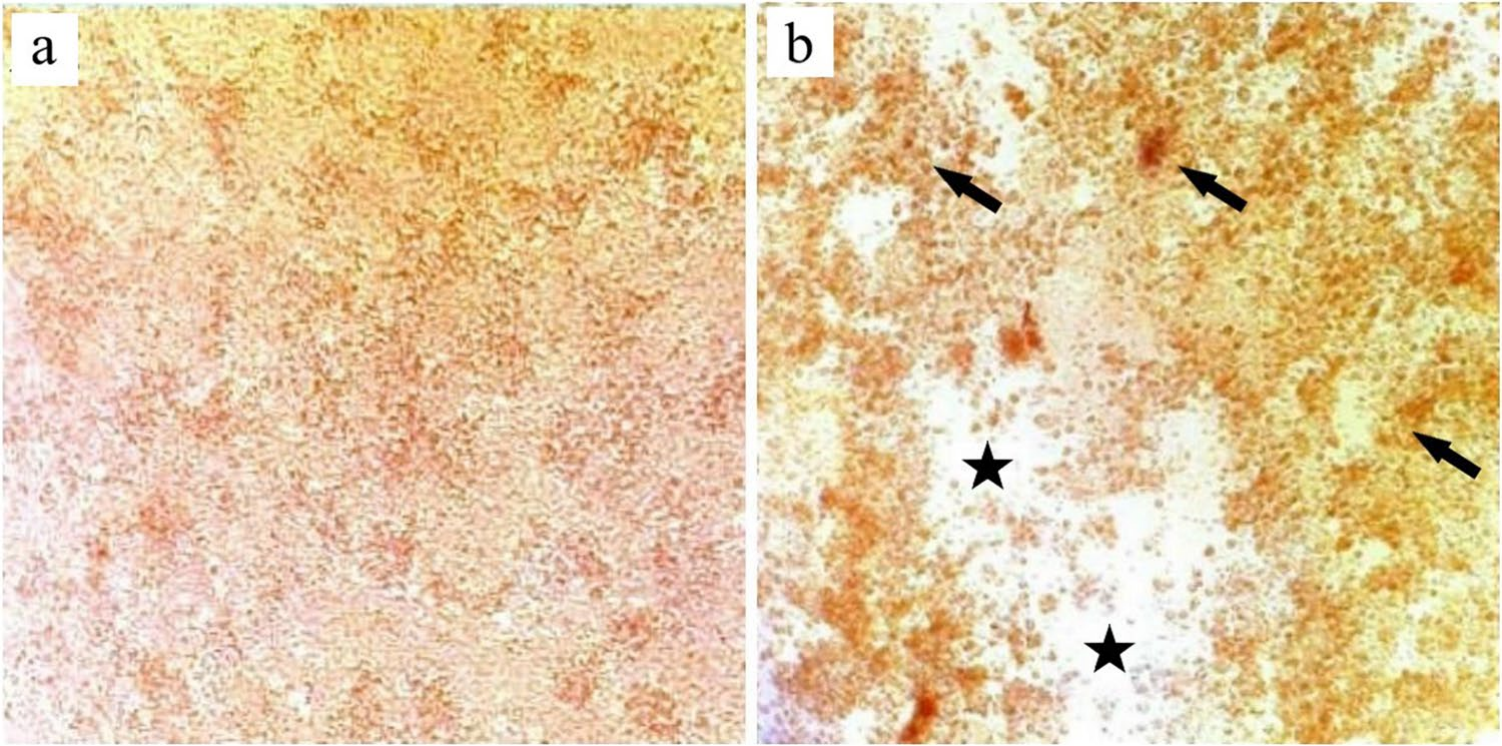
Isolation of CAstV in primary CEL cells. (**a**) Mock-infected CEL cells showing the integral sheet of healthy hepatocytes cells. (**b**) CAstV-infected CEL cells at 72 h post-infection showed small and round cells (with vacuolation), and small regions of the sheet were detached (stars). In addition, aggregation and clustering (arrows) in definite regions of the cell sheet were observed. The CPE represents almost 100% of the cells sheet

Inoculated CEL cells showed aggregation, clustering, vacuolation, and degeneration of hepatocytes. In contrast, the maintained sheet till the first 48 h (Fig. 2). Sloughing of small regions of the cell sheet occurred from the second-day post-inoculation and became more pronounced with the third-day post-inoculation.

#### Sequence analysis

Both Eg/SCU-1CAstV-ORF 1b/2021 and Eg/SCU-2 CAstV-ORF 1b/2021 show complete nucleotide identity to each other, while Eg/SCU-3 CAstV-ORF 1b/2021 and Eg/SCU-4 CAstV-ORF 1b/2021 share 100% identity (Table 2). On the other hand, the samples differ by only 2% nucleotide sequence. Overall, the Egyptian isolates share the highest nucleotide homology (93%) with the Korean isolate Kr/ADL102655-1/2010 and show the most distant relation to the Indian isolate Indovax/APF/1319 with 82–83% homology.

On the level of amino acid sequence, the four Egyptian isolates show the highest homology to the Croatian isolate (CRO-443) with 97% homology (compared to Eg/SCU-1 and Eg/SCU-2) and 100% homology (compared to Eg/SCU-3 and Eg/SCU-4). The Egyptian isolates also shared a high degree of homology with the isolates IR/CAstV/H3778-19/19, MO-SEP-A799-05, and CRO-113 with 96–99%. On the other hand, the Egyptian isolates were most distant from Indian isolate Indovax/APF/1319 with 86–89% homology.

#### Phylogenetic analysis

**Fig. 3:**
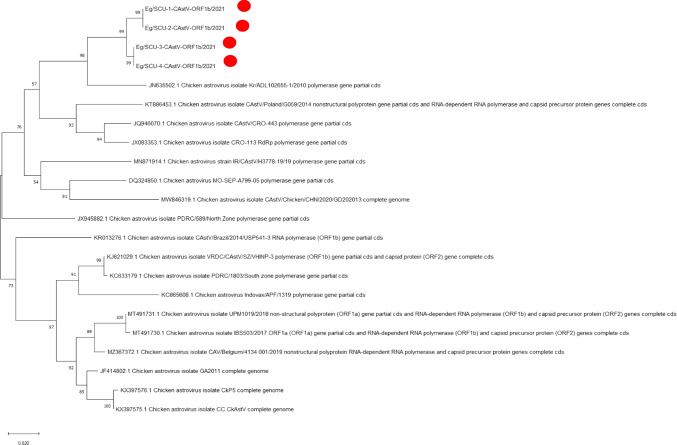
Phylogenetic analysis of the Egyptian CAstV. The phylogenetic tree is based on the partial amino acid sequence of ORF1b (corresponding to RDRP gene). The tree was constructed using MEGA software version X with neighbor-joining using the distance-based methods (1,000 bootstrap replicate). Red circles indicate the isolates of this study

Amino acid sequences were used to create phylogenetic trees to determine the genetic relationship between our Egyptian isolates and 18 reference CAstV strains obtained from GenBank (Fig. 3). Phylogenetic analysis showed that the four Egyptian isolates aligned in close relation to each other. The strains Kr/ADL102655-1/2010, PL/G059/2014, CRO-443, and CRO-113 are the closest strains, and all originated from the same branch. G059/PL/2014 is acknowledged for being under subgroup Aiii. The Egyptian isolates were relatively distant from Chicken astrovirus isolate PDRC/1803/South zone, CkP5, GA2011, and Indovax/APF/1319. These strains belong to subgroup Bii.

### Clinical signs, growth, performance, and mortality rate

#### Clinical signs

**Fig. 4:**
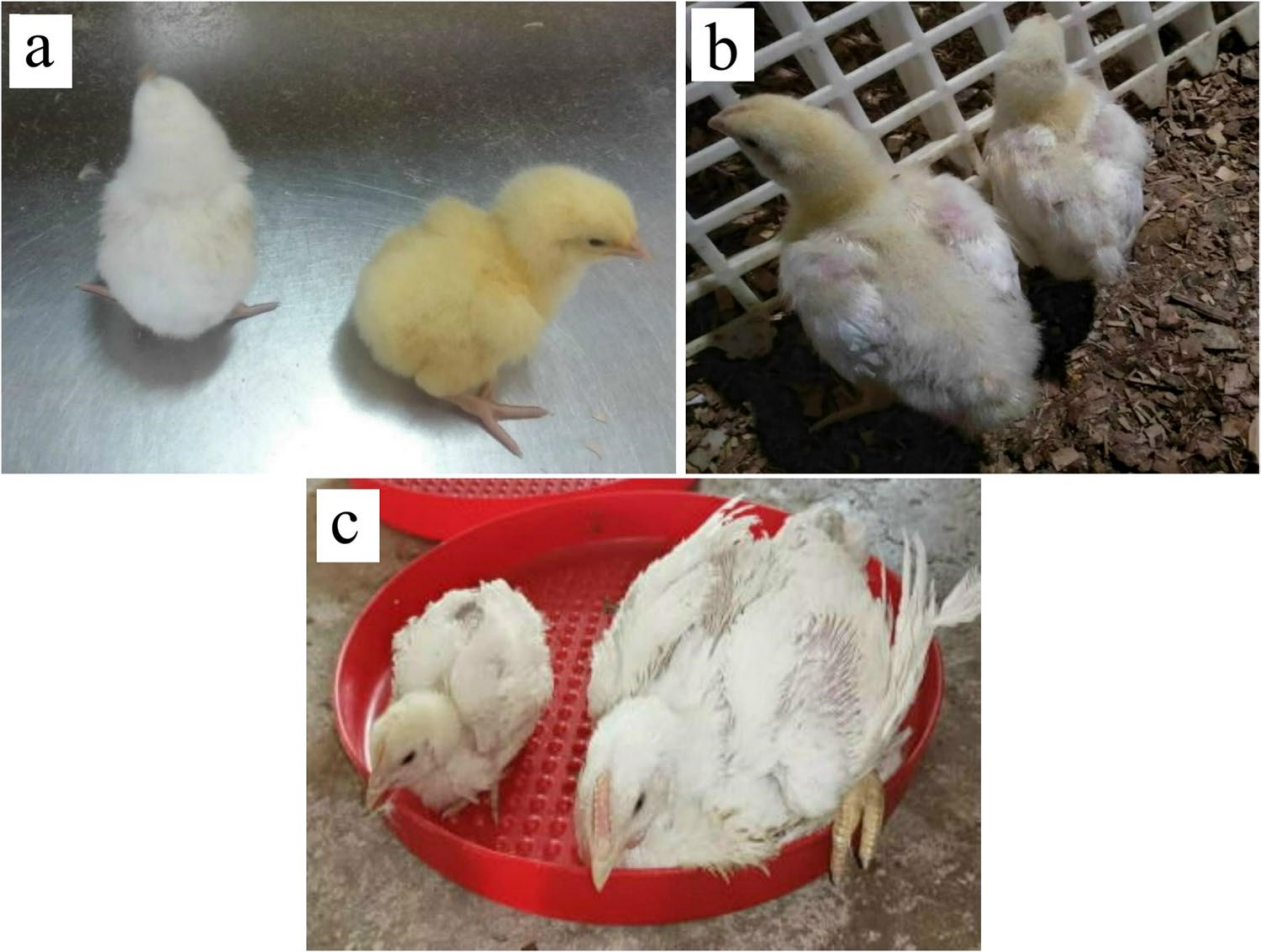
Morphological features of normal and CAstV infected birds (**a**) One-day-old chicks, a stunted white chick is CAstV positive (left), and a normal chick (right) appeared with yellow feathers and good condition. (**b**) 15-day-old chicken, Chicken from CAstV positive flock showing decreased body weight (right) and normal chicken (left) from CAstV negative flock. (**c**) 30 days old chicken, normal CAstV negative chicken (right) and a chicken from CAstV positive flock (left) exhibiting retarded growth

At one day old, CAstV-positive chicks showed characteristic pale to white feathers (Fig. 4) with general weakness and locomotor disorders. Elder chicks on the 15^th^ day exhibited enteric disorders such as diarrhea and retarded growth. Decreased body weight and increased culling percentage were the characteristic features detected on the 30^th^ day of age.

**Fig. 5:**
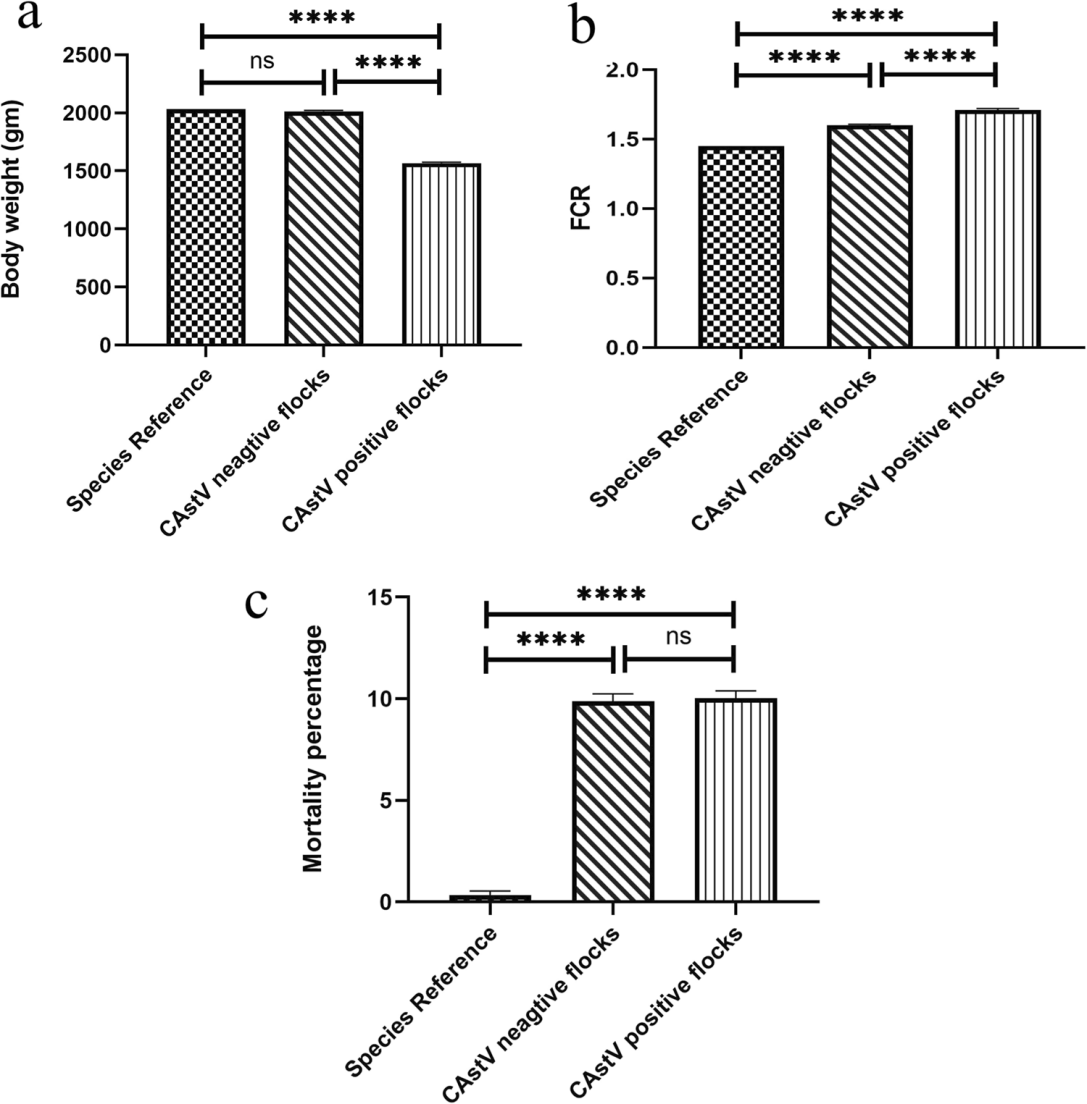
Charts showing (**a**) body weight, (**b**) Feed conversion rate, and (**c**) mortality rate. Data are presented as means ± SEM. Significance was considered at *P*<0.05

Regarding birds' performance (Fig. 5), significantly lower values of estimated body weight were recorded in CAstV-positive flocks compared to normal and species standard values. Similarly, the feed conversion rate was markedly increased in infected flocks. No statistically significant difference was noticed in the mortality rate.

### Gross and histopathological examination

#### Gross findings

On one day old, the most remarkable postmortem findings included livers with bronze to bright green color and pale foci of necrosis. At 15 days old, severe enteritis was detected, as well as hepatitis, swollen kidneys, and mild lung congestion. At 30 days, the same lesions with increased severity were noticed.

#### Histopathological lesions

**Fig. 6:**
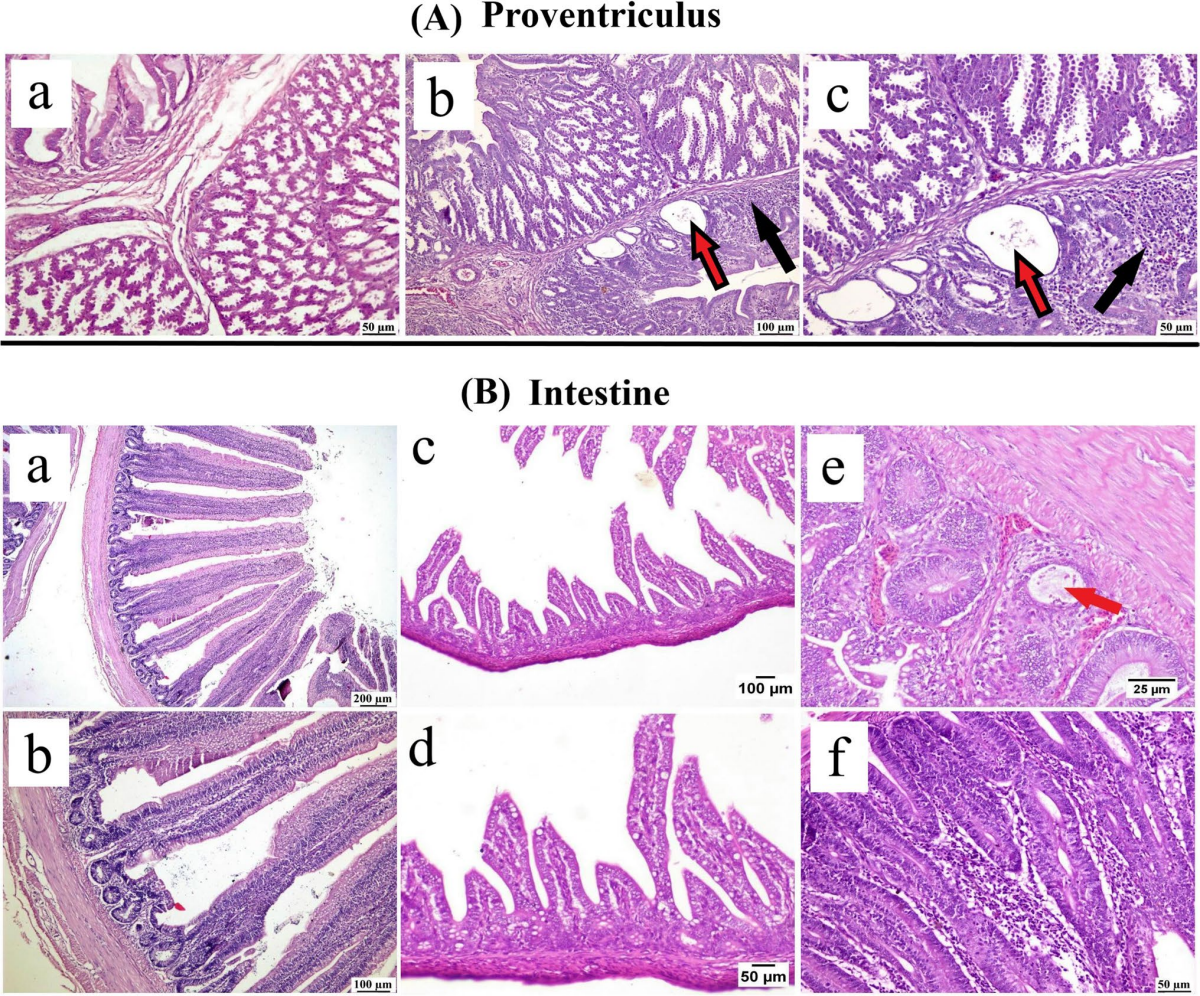
Photomicrographs of (**A**) proventriculus: (**a**) Normal flock: showing normal proventriculus, (**b**) and (**c**) CAstV positive flocks: showing mononuclear inflammatory cells infiltration (black arrow) and cystically dilated glands (red arrow). (**B**) Intestine: (**a**) and (**b**) Normal flocks showing normal intestinal villi. (**c**) and (**d**) CAstV-positive flocks showing short and blunt villi. (**e**) CAstV positive flocks (15 days old), showing dilated crypt with desquamated cells (red arrow), and (**f**) CAstV positive flocks (30 days old), showing intense inflammatory cells infiltration

The proventriculus of CAstV-positive birds (Fig. 6A) showed some changes, including pro ventriculitis at 15 days old with cystically dilated glands that revealed attenuated lining epithelium cells.

Normal (CAstV negative) group showed normal intestine histology with well-oriented villi and intact epithelial lining in all sampling points without any detectable alterations. On the contrary, intestine tissue samples of CAstV-positive chicks exhibited marked histopathological changes (Fig. 6B) ranging from the necrotic epithelial lining on one-day-old chicks, shortening of villi with hyperplastic crypts in 15 days old chicks, accompanied by marked enteritis with hyperactivity of mucous glands at 30 days old chickens.

**Fig. 7:**
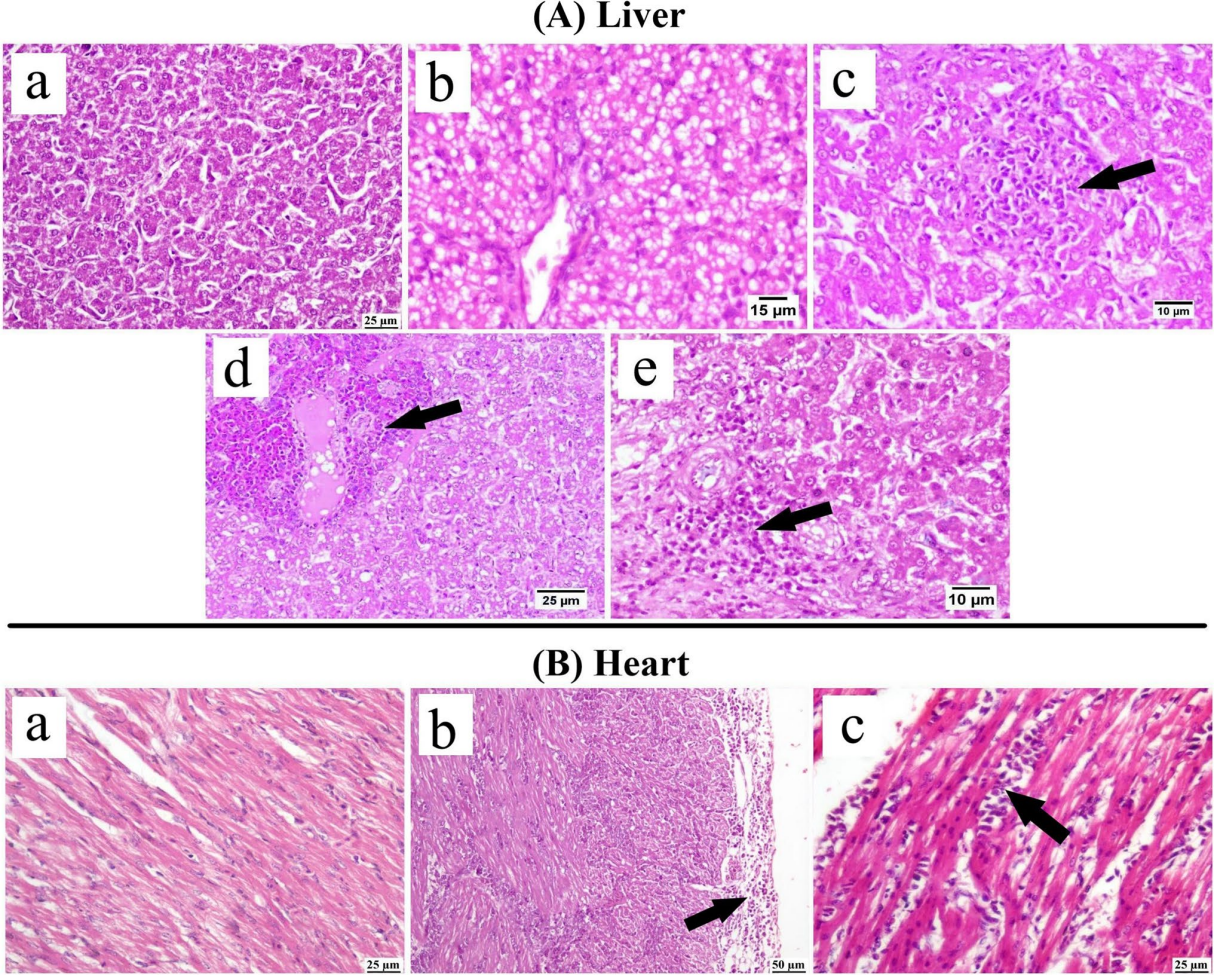
Photomicrographs of (**A**) Liver: (**a**) Normal flocks showing normal hepatic plates, (**b**) CAstV chicks (1 day old) showing marked microvesicular steatosis, (**c**) CAstV chicks (15 days old) showing focal area of hepatocellular necrosis with mononuclear inflammatory cells infiltration (arrow), (**d**) CAstV chickens (30 days old) showing heterophils infiltration (arrow), (**e**) CAstV chickens (30 days old) showing portal infiltration with mononuclear inflammatory cells (arrow). (**B**) Heart: (Photomicrographs) CAstV negative flocks, showing normal myocardium, (**b**) CAstV positive chickens (15 days old) showing pericarditis (arrow), (**c**) CAstV positive chickens (30 days old) showing mononuclear inflammatory cells infiltration in-between muscle fibers (arrow)

Compared to the normal liver (Fig. 7A) detected in CAstV-negative flocks, liver sections from CAstV-infected flocks exhibited marked hepatocellular vacuolation and microvesicular steatosis in one-day-old chicks. Elder birds at 15 days old showed focal areas of hepatocellular necrosis with mononuclear inflammatory cell infiltration. Portal mononuclear and heterophilic cell infiltrations were frequently observed in 30 days old chickens.

The examined heart sections (Fig. 7B) from CAstV-positive chicks revealed pericarditis manifested by intense infiltration of mononuclear inflammatory cells in the pericardium at 15 days old. Myocarditis was noticed in 30 days old birds.

**Fig. 8:**
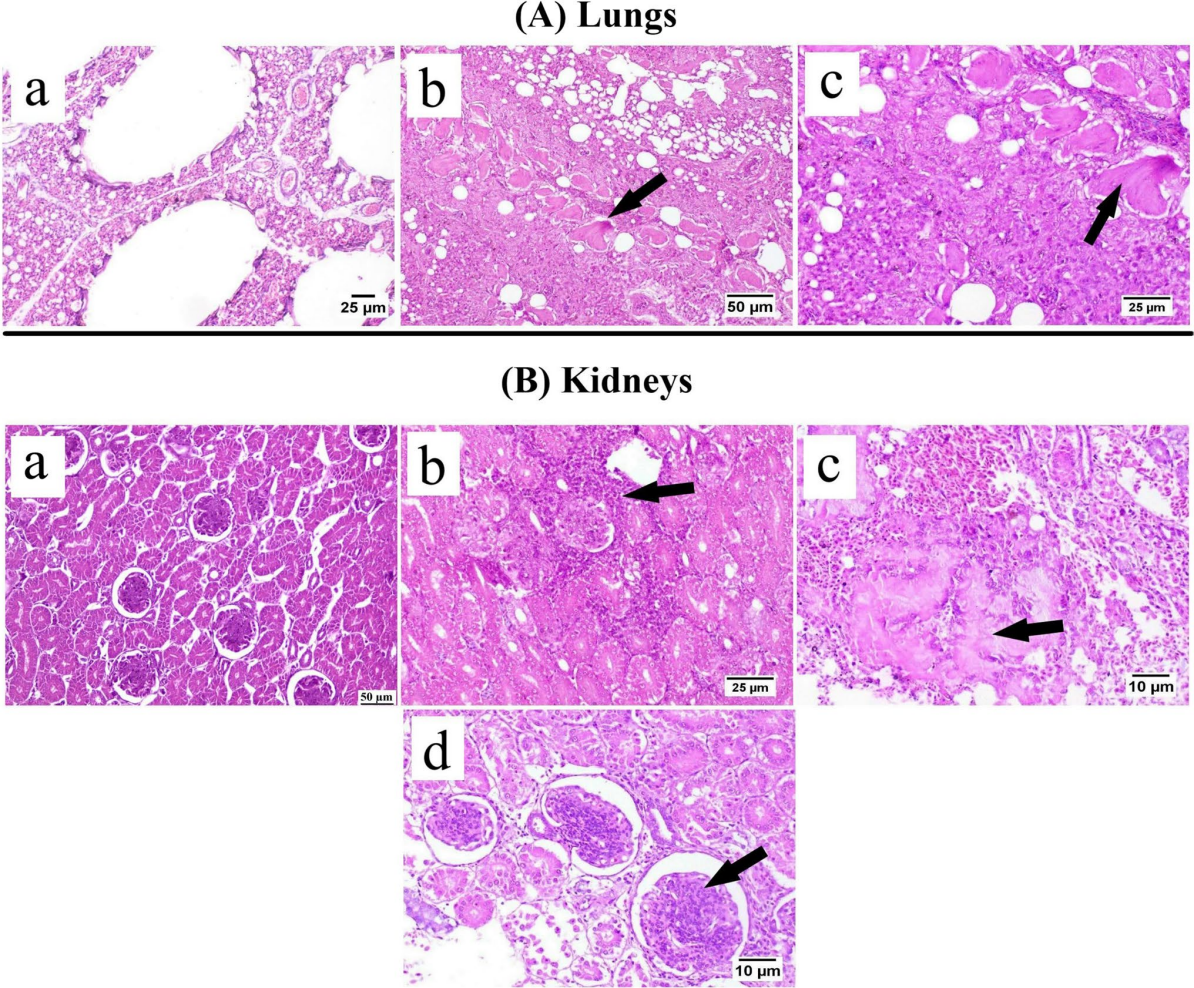
Photomicrographs of (**A**) Lungs: (**a**) CAstV negative chicken showing the normal structure of the lung, (**b**) CAstV positive chicken (30 days old) showing proliferative response with hypertrophy of smooth muscles (arrow), (**c**) higher magnification of previous photo showing hypertrophied smooth muscle (arrow). (**B**) Kidneys: (**a**) CAstV negative chickens showing normal renal tubules and glomeruli, (**b**) CAstV positive chickens (15 days old) showing interstitial infiltration with mononuclear inflammatory cells (arrow), (**c**) CAstV positive chickens (30 days old) showing urate deposition (arrow) with an intense inflammatory reaction, (**d**) CAstV positive chickens (30 days old) showing hypercellularity of glomeruli (arrow)

Microscopic examination of lung tissue sections (Fig. 8A) from CAstV-infected birds (at 30 days old) revealed a proliferative response characterized by hyperplasia of epithelial cells lining parabronchus and hypertrophy of smooth muscles.

Normal renal tubules and glomeruli were detected in CAstV-negative flocks (Fig. 8B). CAstV-infected birds (at 15 days old) showed degeneration and necrosis in the epithelial lining the renal tubules with interstitial nephritis by granulocytes and mononuclear inflammatory cells. Urate deposition with surrounding inflammatory reaction was observed in the kidneys of virus-infected birds at the age of 30 days. Glomeruli showed mesangial-proliferative glomerulopathy characterized by hypercellularity due to the proliferation of mesangial cells in some examined cortical sections.

## Discussion

In the current study, new strains of Chicken Astrovirus (CAstV) were isolated in some broiler chicken flocks associated with retarded growth, performance problems, and marked histopathological changes in different organs, including the intestine, liver, and kidneys.

Phylogenetic analysis showed that Kr/ADL102655-1/2010, PL/G059/2014, CRO-443, and CRO-113 are the closest strains, originating from the same branch. Among these four isolates, PL/G059/2014 is the most well-characterized strain (belonging to the CAst-Aiii group), causing white chicks syndrome and a high mortality rate (Sajewicz-krukowska et al. 2016). In general, white chicks hatchery disease was reported to be associated with CAstV-Bii, -Biv, and -Aiii (Smyth et al. 2013; Long et al. 2018; Palomino-Tapia et al. 2020).

The strains were relatively distant from Chicken astrovirus isolate PDRC/1803/South zone (belonging to CAst-Biii group) and Indovax/APF/1319, both are known to cause Visceral Gout syndrome (Smyth 2017; Panigrahi et al. 2019). Hatchability reduction was reported to be associated with CAstV subgroup Bi and novel chicken astrovirus in China (Zhao et al. 2020; Yin et al. 2021). This suggests that the CAst viruses circulating in Egypt belong to the CAst-Aiii group. However, more isolates need to be isolated to confirm whether other CAst viruses are circulating in Egypt. Noteworthy, previously isolated Middle East CAstV strains were found to share more than 96% amino acid homology with Indian strains, suggesting the circulation of group B-CAstV in the Middle East (Smyth 2017).

Notably, the Korean isolate Kr/ADL102655-1/2010 and the polish isolate PL/G059/2014 were both associated with an unusual mortality rate, among other signs as runting and poor hatchability (Koo et al. 2013; Smyth 2017). On the other hand, the most distant isolate was Indovax/APF/1319. The Indian isolates are known to be predominated by CAstV type Biii (Bulbule et al. 2013; Panigrahi et al. 2019).

Still, ORF2 is more reliable for classification and interpreting genetic relatedness. This work shall be carried out in the further study.

Consistent with our findings, white-colored chicks (WCS) were characteristic for CAstV infection among one-day-old chicks (Schlegel et al. 2016; Sajewicz-krukowska et al. 2016) as well as marked retardation and locomotor abnormalities. Chickens affected by WCS have larger and heavier yolk sacs, indicating decreased yolk consumption, resulting in weak and uncolored chicks (Nuñez et al. 2020).

Mcilwaine et al. described the mechanism by which astrovirus induces white coloration of one-day-old chicks stating that the virus interferes with the transfer of carotenoids into the egg or prevents the embryonic absorption of carotenoids from the yolk sac (Mcilwaine et al. 2021).

As described in our clinical findings, CAstV-infected chickens suffered from enteric signs, including diarrhea, that could be attributed to the different mechanisms by which the virus can induce diarrhea, including modulation of ion balance, destruction of the intestinal epithelium and changing intestinal permeability (Moser and Schultz-Cherry 2005).

Unlike our results regarding the non-significant change in bird mortality, Smyth reported a mortality rate approaching 50% in young birds infected with CAstV; this variation could be regarded as the pathogenicity of the infecting strain and the flock condition (Smyth 2020).

In agreement with our gross findings, Nuñez et al. mentioned white feathers of one-day-old chicks, enlarged liver with greenish discoloration and necrotic foci, enteritis, and swollen kidneys as the main macroscopic findings of CAstV-infected birds (Nuñez et al. 2020).

Histopathological study revealed necrosis of intestinal epithelial lining and shortening and blunting of villi with hyperplastic crypt; these findings were in agreement with those previously described by (Moser and Schultz-Cherry 2005; Sellers et al. 2009).

Chicken villous epithelium are initially susceptible to CAstV infection, but later the virus became able to spread and replicate in the crypts of Lieberkühn that actively divide, and the virus migrates toward the villi (Kang et al. 2018).

Our described kidney lesions were frequently described in previous studies (Wit et al. 2011; Bulbule et al. 2013), including degeneration of renal tubules with interstitial nephritis and urate deposition.

CAstV infection was also associated with lesions in different organs, including hepatocellular degeneration and necrosis, myocarditis, and pulmonary congestion (Bulbule et al. 2013).

## Conclusions

CAstV infection represents a challenge facing poultry production by affecting the growth, feed conversion rate, and bird performance. Early detection and frequent screening of breeder flocks are required to maintain the flock's health and to ensure the highest performance.

## Supplementary Information

Below is the link to the electronic supplementary material.

Supplementary file1 (TXT 11 KB)

## Data Availability

“The datasets generated during and/or analysed during the current study are available from the corresponding author on reasonable request.”
